# Supplementary figures and images for: The impact of COVID-19 and control measures on public health in Thailand, 2020
Source: PeerJ. 2022 Feb 16;10:e12960. doi: 10.7717/peerj.12960 (PMC8857899; doi:10.7717/peerj.12960)

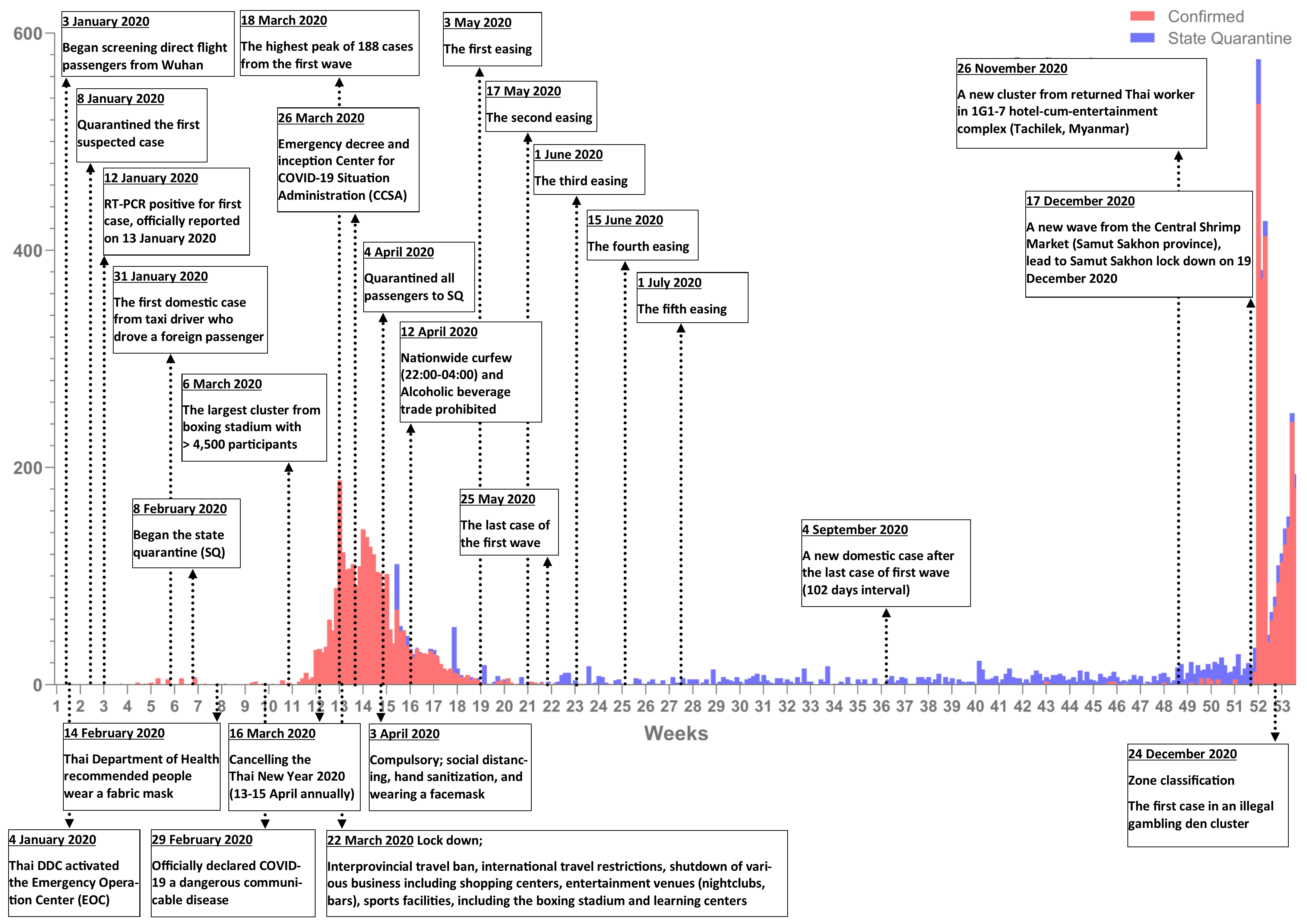

Supplement: Supplemental Information 1 [file peerj-10-12960-s001.png]
